# Normalizing Dietary Supplement Product Names Using the RxNorm Model

**DOI:** 10.3233/SHTI190253

**Published:** 2019-08-21

**Authors:** Jake Vasilakes, Yadan Fan, Rubina Rizvi, Anusha Bompelli, Olivier Bodenreider, Rui Zhang

**Affiliations:** a Institute for Health Informatics, University of Minnesota, Minneapolis, MN, USA; b Department of Pharmaceutical Care & Health Systems, University of Minnesota, Minneapolis, MN, USA; c Lister Hill National Center for Biomedical Communications, National Library of Medicine, National Institutions of Health, USA

**Keywords:** Dietary supplements, RxNorm, Natural Language Processing

## Abstract

The use of dietary supplements (DSs) is increasing in the U.S. As such, it is crucial for consumers, clinicians, and researchers to be able to find information about DS products. However, labeling regulations allow great variability in DS product names, which makes searching for this information difficult. Following the RxNorm drug name normalization model, we developed a rule-based natural language processing system to normalize DS product names using pattern templates. We evaluated the system on product names extracted from the Dietary Supplement Label Database. Our system generated 136 unique templates and obtained a coverage of 72%, a 32% increase over the existing RxNorm model. Manual review showed that our system achieved a normalization accuracy of 0.86. We found that the normalization of DS product names is feasible, but more work is required to improve the generalizability of the system.

## Introduction

Dietary supplements (DSs) are defined as “products taken by mouth that contain a dietary ingredient that includes vitamins, minerals, amino acids, and herbs/botanicals, as well as other substances that can be used to supplement the diet” [1]. They comprise one of the fastest growing categories of complementary and alternative medicines [2]. According to the National Health and Nutrition Examination Survey (NHANES), the age adjusted consumption of DSs has steadily increased, both in male (28% to 44%) and female (38% to 53%) populations [3], especially among adults aged ≥60 years where 70% have reported using one or more DS [4]. Increasing usage of DSs has led to substantial market growth resulting in wide availability of dietary supplement products.

The regulations covering DSs are much less stringent than those covering commonly consumed foods and clinical drugs [1], even though DS adverse events and DS-drug interactions are common [5, 6] and potentially severe [7]. DS products and dietary ingredients are regulated by the U.S. Food and Drug Administration (FDA) under the Dietary Supplements Health and Education Act (DSHEA). As part of this, the FDA developed guidelines to help ensure that DSs sold in the United States (produced both domestically and abroad) are properly labeled. However, according to the FDA, “those guidance documents only represent the agency’s current perspective and should be viewed only as recommendations, unless specific regulatory or statutory requirements are cited” [8, 9]. Thus, it is not required to obtain approval of a label in order to import or distribute a DS, and failing to comply with the guidelines does not entail any legally enforceable consequences.

To make matters worse, DS product names express ingredient and brand information in a large variety of ways. Product names often include additional components such as ingredient qualifiers (e.g. “leaf”, “dried”, “extract”), dose information (e.g. “capsules”, “10mg”), and flavors. This, along with loose labeling guidelines, have resulted in DS product names that lack a consistent structure, which hinders critical tasks such as cross-platform communicability and the reuse of DS knowledge.

The situation is very different for clinical drugs. In addition to stricter regulations regarding drug naming, the U.S. National Library of Medicine develops RxNorm, a normalized naming system for generic and branded drugs [10]. It supports semantic interoperability between sixteen drug terminologies and pharmacy knowledge bases. RxNorm normalizes drug names using a set of 15 term types corresponding to drug entities [11]. Term types are codes which indicate the level of specificity of a given drug name or qualifier. For example, the drug name “Fluoxetine” is assigned the term type *IN* (ingredient) and the qualifier “Oral solution” is assigned *DF* (dose form). Some RxNorm term types are the combination of two or more atomic term types. For example, the *IN* and *DF* term types combine into *SCDF* (Semantic Clinical Drug Form), such as in “Fluoxetine Oral Solution”.

As a means of normalizing drug names, RxNorm plays an essential role in decision support, quality assurance, healthcare research, reimbursement, and mandatory reporting [12]. Similarly, normalizing DS product names is an important step. By providing a reliable way to refer to DS products, it would facilitate DS pharmacovigilance and knowledge discovery such as in [13, 14]. However, Y. Wang et al. showed that existing normalization resources such as RxNorm and UMLS cover only a fraction of DS terms, indicating a need for DS-specific resources [15]. Sharma and Sarkar developed such a resource to extract DS mentions from adverse event reports and clinical notes, but their system is restricted to ingredients and do not consider related concepts such as dose form or strength, which are crucial in the RxNorm model [16, 17]. A recent study by L. Wang et al. showed promising results applying and extending the RxNorm model to Chinese clinical drugs [18]. In a similar vein, this study evaluates the feasibility of applying an RxNorm-like normalization approach to DS product names. We developed a rule-based natural language processing (NLP) system which is able to find various components of the product names and assign them to term types, which can be used for normalization. Our system leverages three existing terminologies to develop the NLP patterns: The Therapeutic Goods Administration (TGA) [19], RxNorm, and iDISK - an integrated knowledge base of DSs and related terms [20]. We evaluated the generalizability of the system by reporting its coverage and accuracy on a set of product names extracted from the Dietary Supplement Label Database (DSLD) [21].

## Methods

**Figure 1–:**
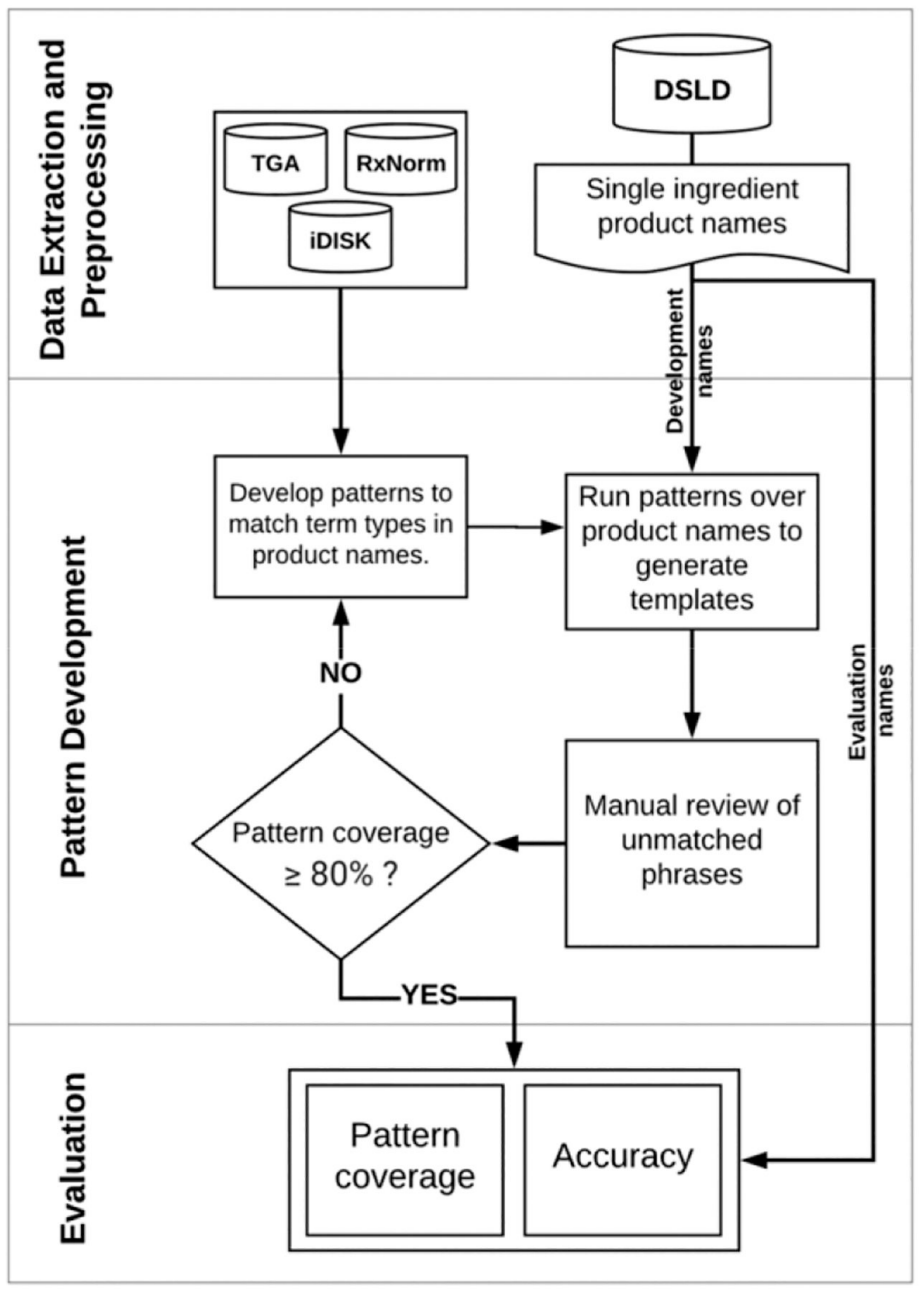
The study design.

This study is comprised of three phases: data extraction and preprocessing, NLP pattern development, and evaluation. Figure 1 illustrates the overall process and each phase is detailed below.

### Data Extraction and Preprocessing

We extracted 12,383 product names from DSLD using a web scraper. We restricted the extracted names to those listed as containing a single dietary ingredient in order to reduce the amount of variability in the product names. This set of names was then split into a development set (9,906, 80% of the original data) and evaluation set (2,477, 20% of the original data). In order to ensure both sets were representative of the full data set, the split was stratified on the LanguaL (http://www.langual.org) product type assigned to the DS product by DSLD.

### NLP System Development

The development set of product names was used to build the NLP system. This system was built using an iterative process comprised of four stages:

1. We developed a set of term types corresponding to components of the product names, detailed in Table 1. We also developed regular expression patterns to match these term types in the product names. These patterns used keyword lists obtained from TGA and RxNorm to match components such as dosages and dose forms, plant preparations (e.g. dried leaf), etc, as detailed in Table 1. We used the ingredient name thesaurus from the iDISK knowledge base to match ingredient names as well as a regular expression for certain vitamins. Where necessary, we manually augmented these keywords lists with lexical variants such as abbreviations and plural forms (e.g. “cap” and “capsules” in addition to “capsule”). Brand names were matched using a combination of a rule based method and manually curated list of brand names extracted from the development set. We removed common stop words from the product names in addition to defining a stop word term type (*STOP*) in order to designate which words should not be included in the normalized product names.
2. For each name in the development set, we searched for each pattern in turn. Thus the output of this step is an ordered list of term type codes each corresponding to a matched span in the product name string. The ordering of this list matches as closely as possible the RxNorm term types. For example, running the patterns on the product name “Herb Pharm Elderberry” returns the list of matched term types *BN IN*, where *BN* (brand name) matches “Herb Pharm” and *IN* (ingredient) matches “Elderberry”. We call each unique list of term types a template. Note that the *STOP* term type is not included in the final templates. Templates correspond to the higher-level RxNorm term types such as *SCDF*. Ambiguous contexts were handled either by the regular expressions themselves (e.g. “mg” for milligrams must be preceded by a number to avoid confusion with magnesium), or by the order in which the patterns were searched. Regarding the latter case, brand names were search first, followed by ingredients, as these have the most potential for overlap with other term types.
3. We computed the coverage of the patterns on the development set. This included the number of fully matched (i.e. all parts of the name were matched to one or more patterns), partially matched (i.e. some substring of the name was matched), and unmatched product names. Our target full-match coverage on the development set was 80%. If our system did not reach this target, we reviewed the partially matched and unmatched names (step 4 below) and proceeded with the next round of development. If it met or exceeded 80% we moved on to evaluation.
4. At each iteration of the pattern development cycle while the full-match coverage was below 80%, Two health informaticians (RR and AB) manually reviewed 20% of the partially matched and unmatched product names. The results of this review were used to modify existing patterns and create new patterns to improve the coverage of the system.

### Evaluation

Evaluation proceeded after our pattern matching system obtained the target 80% full-match coverage on the development set. At this point we ran the pattern matching system on the 2,477 held out evaluation product names and computed the coverage on the evaluation set. Each fully matched product name corresponds to a template which is output by our system. For each template that is also present in RxNorm, we report the frequency with which it occurred in the development and evaluation sets.

Additionally, we evaluated the accuracy of our system on the evaluation set in two ways:

1. We measured the accuracy of the term type patterns on the evaluation set. Each *matched span - term* type pair in each product name in the evaluation set was annotated according to its correctness. We assigned a 1 if the words within the span belonged to the corresponding term type, or a 0 otherwise. We then computed the accuracy for each term type using these annotations.
2. We measured the accuracy over the product names in the evaluation set. This was computed by averaging the accuracies of the product names, where the accuracy of a given product name n is the mean of the labels assigned to each token in the name in step 1, computed by
  $$accuracy\left(n\right)=\frac{1}{\left|T\left(n\right)\right|}\underset{t\in T\left(n\right)}{\sum }l\left(t\right)$$
  Where *T*(*n*) is a function that returns the tokens in the product name *n* and *ℓ*(*t*) is a function that returns the label (1 or 0) for token *t*.

In the case of partially matched or unmatched product names, each unmatched token is implicitly assigned a 0. This allows us to compute the accuracy of partially matched and unmatched product names and thus an accuracy value for the entire evaluation set.

## Results

Running the pattern matching system over the development set produced 129 unique templates using all 13 term types after removing *STOP*. Running the system on the evaluation set produced 62 unique templates, 7 of which were not seen in the development set, for a total of 136 templates. The *TIME* term type was not present in any full matches on the evaluation set. The 5 most frequent templates across the development and evaluation sets are shown in Table 2.

8 of the 129 development templates and 5 of the 62 evaluation templates matched existing RxNorm term types. The frequencies of these templates in the development and evaluation sets are given in Table 3. In both the development and evaluation sets the *BN IN STR* template (*SBDC* in RxNorm) accounted for about one third (33%) of the fully matched product names. The most frequent of these templates are also the first, third, and fourth most frequent templates overall in both the development and evaluation sets, shown in Table 2. Note that the second most frequent template, *BN IN*, does not have a corresponding RxNorm term type.

The coverage of the final NLP system on the evaluation set, after obtaining 80% full match coverage on the development set, was 71.9% full match, 27.6% partial match, and 0.5% unmatched. Thus only 11 (0.5%) evaluation product names were completely unmatched by our system. Compared to the coverage of RxNorm term types (39.89%), our system improves full-match coverage by 32% on the evaluation set.

Table 4 shows the average accuracy of the pattern matching system on the evaluation product names. The average of the fully matched names is 0.30 greater than the partially matched names. This is expected due to the fact that each unmatched token in the partially matched names is treated as incorrect. Still, because the majority of the names in the evaluation set were fully matched, the average accuracy (0.86) is closer to the fully matched accuracy.

The accuracy of each term type, computed over the fully matched and partially matched evaluation set names, is given in Table 4. We report both the average accuracy of each term type over all the evaluation names (given by the bars) as well as the accuracy on the fully matched and partially matches names separately (given by the triangles and Xs, respectively).

A number of term types achieved a perfect 1.00 accuracy, and the most common term types, *BN* and *IN*, achieved accuracies above 0.90. *ANM* (animal source) obtained the lowest accuracy, with 0.61.

## Discussion

As shown in Table 3, only about 40% of DS product names fit existing RxNorm term types. This suggests that RxNorm is not well suited to the space of DS products. Table 2 and Table 3 show that brand names (*BN*) play a significant role in DS product labeling, with most or all of the most frequent templates containing *BN*. Indeed, further investigation revealed that 91% of the patterns generated on the development and evaluation sets contain *BN*. This is not surprising, given the different ways in which drugs and DS products are marketed. Drugs are carefully prescribed and regulated, with brand name and generic drugs being for the most part interchangeable, meaning that the *IN* term type is most useful for clinicians, patients, and regulators. On the other hand, there are many competing DS products containing similar ingredients so DS manufacturers emphasize product branding to appeal to consumers.

Nevertheless, the accuracy of our system indicates promising potential for normalizing DS product names. Even when treating unmatched words as misses, our system was able to achieve an accuracy of 0.86 on the evaluation set, which improves to 0.95 on fully-matched product names only (Table 4).

On the other hand, the generalizability of our system is limited by its coverage. Our system was only able to fully match 71.9% of the evaluation set names, a difference of 8% from the development set coverage (80%). Still, a majority of the remaining names were partially matched (27.6%) and our system was unable to find a match for only 11 (0.5%) of the evaluation names.

Reviewing examples of unmatched words in the development and evaluation sets revealed that many were unseen brand names. For example, our system missed the brand name “Cellucor COR-Performance” because it did not occur in the development set. In the future, the use of machine learning methods could improve the coverage and accuracy of the system on brand names, which are too numerous and varied to be manually curated. Many other unmatched words were uninformative buzzwords such as “High Intensity Training Program”. We found that most errors for the *IN* term type were due to the inclusion of phrases that belong to *PLNT* or *PREP*, such as in “Peppermint *Leaf*” and “Green Tea *Extract*”. These occurred because our system searched the *IN* patterns before *PREP* and *PLNT* and the iDISK ingredient thesaurus often includes these phrases in ingredient names. Also, many brand names contain implicit information regarding claims or ingredients which our system could not match. For example, “PomGuard” in “Jarrow Formulas PomGuard” suggests the inclusion of pomegranate as in ingredient.

This study has the following limitations: First, we only include single ingredient products from DSLD. Single ingredient products comprise 22% of all product names extracted from DSLD, so the generalizability of our method to multi-ingredinet products remains to be investigated in future work. Second, because of the above limitation, we assume the presence of at most one ingredient in each product name. Still, some product names listed as single ingredient in DSLD contain more than one ingredient mention, e.g. “Physician’s Preference *Royal Garlic* with *Hawthorn* and *Cayenne*”. It would be straightforward to modify our system to search for multiple ingredient mentions, which would increase coverage. Third, our system has limited ability to disambiguate context.

Therefore, only one meaning was chosen for any polysemous keywords, e.g. keywords that occurred in more than one TGA list. Important future work would be to employ more advanced NLP and machine learning methods to disambiguate context in product names. This could vastly improve the accuracy of term types such as ANM, which contains keywords (e.g. “Heart” and “Liver”) that are often confused with claims or uses.

## Conclusions

In this study we developed and evaluated an NLP system to apply an RxNorm-like normalization approach to dietary supplement product names. As has been done for drugs, normalization is important to facilitate interoperability and the search for information about DSs. We found that the existing RxNorm drug normalization templates do not generalize to dietary supplements and that it is necessary to extend the RxNorm model to sufficiently cover DS product names. The normalization system outlined here obtains a substantial increase (32%) in coverage on DS product names over RxNorm as well as an accuracy of 0.86. Nevertheless, there is great variability in supplement product names and more work is required to improve the performance of our system.

## Figures and Tables

**Figure 2–:**
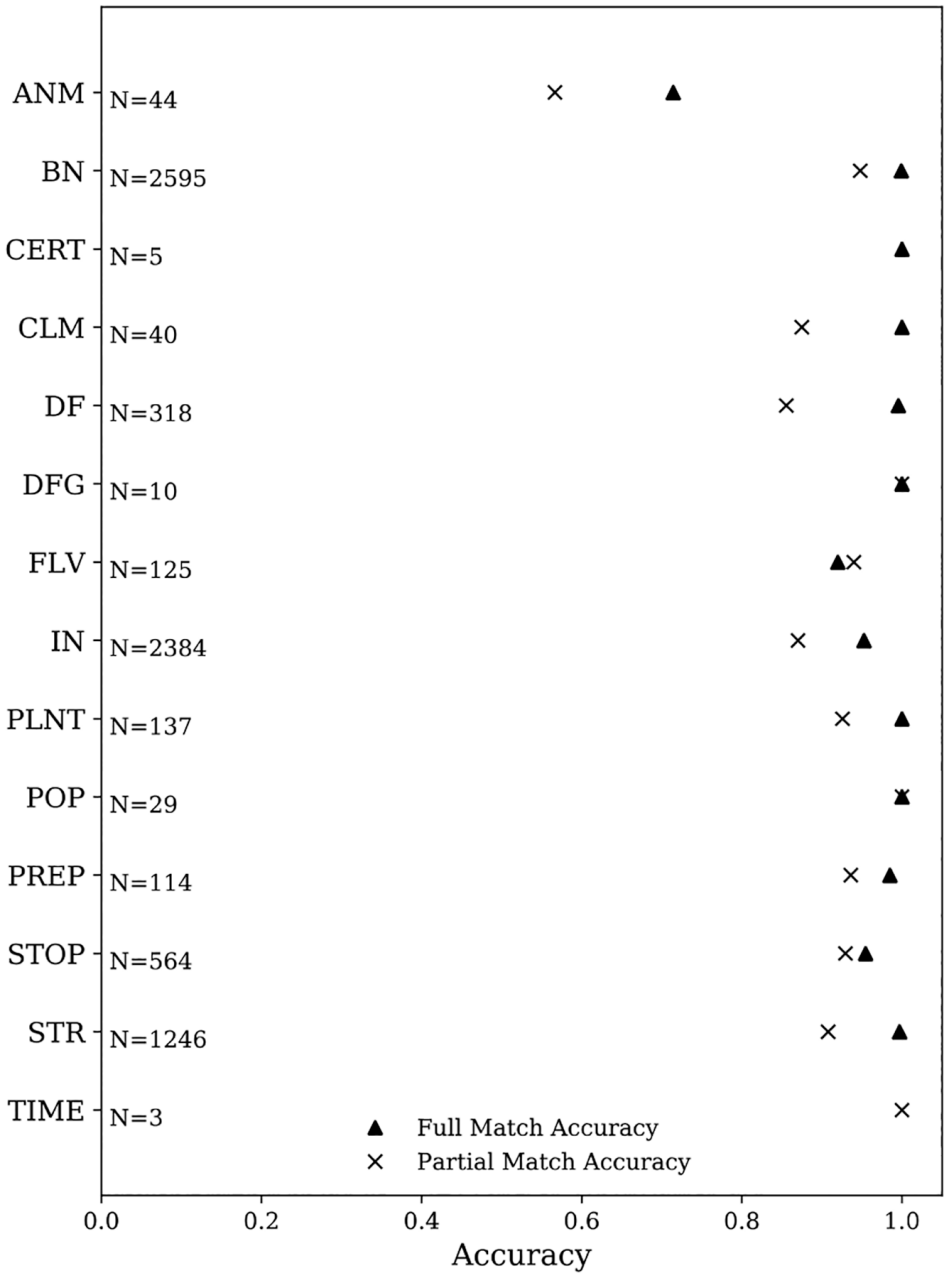
Accuracy of each term type on the evaluation set. The bars indicate the average accuracy of the term types over the full and partially matched product names in the evaluation set. The points indicate the accuracy on the fully and partial matched names, respectively.

**Table 1–:** Term types used in the product name normalization system.

Term Type (Abbreviation)	Description	Example	Pattern Source
Animal Source (ANM)	The part of an animal from which the ingredient is derived.	Bone Marrow	TGA
Brand Name (BN)	Manufacturer’s name.	GNC	Annotation, rules
Certification (CERT)	Official certifications claimed by the product.	USP certified	TGA
Claim or Use (USE)	A description of the purported use of a dietary supplement.	Sleep aid	Annotation
Dose Form (DF)	The phsyical from of the product.	Capsule	TGA, RxNorm
Dose Form Group (DFG)	A grouping of dose forms related by route of administration.	Topical	TGA, RxNorm
Flavor (FLV)	The flavor of a supplement.	Strawberry	Annotation
Ingredient (IN)	Name of the dietary supplement ingredient.	Gingko Biloba	iDISK, rules
Plant Source (PLNT)	The part of a plant from which the ingredient is derived.	Leaf	TGA
Demographic or Population (POP)	The group of persons for whom the product is intended.	Children’s	TGA
Preparation (PREP)	A descriptor of how an ingredient is prepared.	Dried	TGA
Stop Word (STOP)	Uninformative words that are to be excluded from the normalized form.	With, Natural	Annotation
Strength (STR)	The quantity of the ingredient in a product.	100 mg	TGA
Time of Use (TIM)	When the product is intended to be used.	Night time	TGA

**Table 2–:** The 5 most common templates and their product name coverage across the development and evaluation sets along with examples for each.

Frequency ranked templates	Example product name
BN IN STR (32.0%)	Bronson Laboratories Vitamin E 200 IU
BN IN (21.3%)	NutraBio Melatonin
BN IN DF (3.4%)	TERRAVITA Potassium Citrate Powder
BN IN STR DF (3.0%)	Optimum Nutrition Tribulus 625 MG Caps
BN IN PLNT (1.9%)	Nature’s Answer Hawthorn Berry

**Table 3–:** Frequencies of templates generated on the development and evaluation sets that match RxNorm term types, computed using the fully matched product names. We do not include the following RxNorm term types: Precise Ingredient (PIN), Multiple Ingredients (MIN), Generic Pack (GPCK), Brand Name Pack (BPCK) as they are not applicable to this study.

RxNorm Term Type	Corresponding Template	Dev Frequency	Eval Frequency
Ingredient (IN)	IN	1 (0.01%)	1 (0.04%)
Semantic Clinical Drug Component (SCDC)	IN STR	1 (0.01%)	0
Semantic Clinical Drug Form (SCDF)	IN DF	2 (0.02%)	0
Semantic Clinical Dose Form Group (SCDG)	IN DFG	0	0
Semantic Clinical Drug (SCD)	IN STR DF	3 (0.03%)	0
Brand Name (BN)	BN	209 (2.11%)	10 (0.40%)
Semantic Branded Drug Component (SBDC)	BN IN STR	3353 (33.85%)	812 (32.78%)
Semantic Branded Drug Form (SBDF)	BN IN DF	370 (3.74%)	80 (3.23%)
Semantic Branded Dose Form Group (SBDG)	BN DFG	0	0
Semantic Branded Drug (SBD)	BN IN STR DF	325 (3.28%)	85 (3.43%)
**Total**		**4264 (43.04%)**	**988 (39.89%)**

**Table 4–:** Overall accuracy on the evaluation set, reported for all evaluation names, only those which were fully matched, and only those that were partially matched.

Match Type	Accuracy
Full + Partial + None	0.86
Full match only	0.95
Partial match only	0.65
